# Long‐Term Clinical Response to Medical Treatment, Behavioral Therapy, or Their Combination in Cats With Hyperesthesia Syndrome

**DOI:** 10.1111/jvim.70174

**Published:** 2025-06-17

**Authors:** Claudia Pauciulo, Stefania Uccheddu, Andrea Corda, Federica Biggio, Daniele Sebastian Corlazzoli, Marika Menchetti, Antonella Gallucci

**Affiliations:** ^1^ Veterinary Neurology Center Rome Italy; ^2^ Behavioral Medicine and Animal Welfare Division, San Marco Veterinary Clinic and Laboratory Veggiano Italy; ^3^ Department of Veterinary Medicine Veterinary Teaching Hospital, University of Sassari Sassari Italy; ^4^ Veterinary Neurological Center “La Fenice” Selargius Italy; ^5^ Neurology and Neurosurgery Division San Marco Veterinary Clinic and Laboratory Veggiano Italy

**Keywords:** behavioral modification, behavioral therapy, cats, feline, feline behavior, feline neurology, fluoxetine, hyperesthesia syndrome, rolling skin

## Abstract

**Background:**

Hyperesthesia syndrome (HS) is a common yet poorly understood condition in cats, with hypothesized neurological and behavioral causes and limited data on outcomes and treatment.

**Objectives:**

This study aimed to describe the clinical outcomes and the treatment response of 28 cats with HS, managed through various therapeutic strategies.

**Animals:**

Clinical records of 28 cats with HS and minimum of 1‐year follow‐up were reviewed.

**Methods:**

Retrospective, observational, descriptive study conducted on a case series of cats affected by HS. Sixteen cats (57%) received fluoxetine alone (Fluoxetine‐only), seven (25%) were managed with behavioral modification and gabapentin or fluoxetine (Fluoxetine/Gabapentin + Behavior) and five (18%) were treated with behavioral modification alone (Behavior‐only).

**Results:**

An episode‐free period (EFP) ≥ 9 months was observed in 23 (82%) cats. Fifteen cats (94%) in the Fluoxetine‐only group experienced an EFP of ≥ 9 months. Moreover, they had a shorter time to recovery (median [IQR] = 8 [3.5–18] days) compared to the Fluoxetine/Gabapentin + Behavior and Behavior‐only groups (median [IQR] = 100 [90–210] and 60 [30–90] days, respectively). At the 1‐year follow‐up, 26 (93%) cats no longer had HS clinical signs and 14 (50%) were still under pharmacotherapy. Relapses were reported only in one case (4%).

**Conclusions:**

Most of the cat's diagnosed with HS, and managed through various therapeutic strategies, experienced an EFP of more than 9 months and showed absence of clinical signs at 1 year follow‐up.

**Abbreviations**
CSF: cerebrospinal fluid
ECAWBM: European College of Animal Welfare and Behavioral Medicine
ECVN: European College of Veterinary Neurology
EFP: episode‐free period
GABA: gamma‐aminobutyric acid
HS: hyperesthesia syndrome
IQR: interquartile range
MRI: magnetic resonance imaging
SSRIs: selective serotonin reuptake inhibitors

## 1 Introduction

Hyperesthesia syndrome (HS) is a condition of cats characterized by episodic lumbar skin hypersensitivity with rolling or rippling, sudden bursts of activity with jumping, and potential self‐trauma. It was first identified in 1980, as “atypical neurodermatitis,” “rolling skin syndrome,” or “twitchy cat disease” [1]. Since then, the main clinical signs observed have included licking or biting the flank and lumbar areas; muscle spasms in the dorsal lumbar region, as well as in the tail and anal areas; and a rippling motion of the skin. In severe cases, cats might self‐mutilate their tails, requiring prompt medical intervention [2, 3, 4]. Additional clinical signs associated with HS include vocalizations, episodes of sudden jumping and running, apparent hallucinations, and behavioral changes [1].

For these reasons, potential causes of HS have included seizure disorders, such as epilepsy, idiopathic, or secondary to structural diseases such as encephalitis and brain tumors [2, 3, 5, 6]. Given the lumbar region involvement, etiological hypotheses have also explored spinal conditions with neuropathic pain such as intervertebral disc disease, infectious myelitis, and spinal neoplasia [2, 3].

Hyperesthesia syndrome is associated with dermatological conditions, such as hypersensitivity dermatitis, or with behavioral issues (compulsive disorders and episodes such as displacement behaviors, triggered by any period of physiological, or psychological hyperarousal) or orthopedic problems, such as tail trauma [2, 3, 7].

Because of this uncertainty, past treatments have varied widely. Considering possible epileptic causes, medications such as phenobarbital and diazepam have been administered in the past [1].

Other studies have combined anti‐inflammatory drugs such as prednisolone with adjuvant analgesics (primarily gabapentin), as well as behavior‐modifying medications (e.g., lorazepam, oxazepam, buspirone, amitriptyline, fluoxetine, paroxetine, or clomipramine), a synthetic progestin (megestrol acetate), and various vitamins and supplements (such as acetyl‐L‐carnitine, coenzymes, riboflavin, and vitamin E) [1, 2, 3, 8, 9].

A previous study suggested that HS in cats could be a behavioral displacement disorder, which led to the hypothesis that HS signs might decrease after behavioral modification or treatment with psychoactive drugs such as selective serotonin reuptake inhibitors (SSRIs), tricyclic antidepressants, or glutamate‐modulating drugs [2, 9]. Behavior displacement disorder is an activity manifested out of context as the animal is prevented from or unable to enact another behavioral response or occupy itself [10]. Therefore, neurological and behavioral causes have actually been considered the primary underlying mechanisms of the condition.

To the authors knowledge, the literature lacks information on the long‐term outcomes of pharmacotherapy and behavioral modification in cats affected by HS.

The aim of the present study was to describe the 1‐year clinical outcomes in a case series of cats with HS, treated with different therapeutic approaches.

## 2 Materials and Methods

Retrospective, observational, descriptive study. Medical records of cats referred to the Veterinary Neurological Center “La Fenice” (Selargius, Italy) and the Veterinary Clinic and Laboratory “San Marco” (Veggiano, Italy) between January 2018 and December 2023 with a diagnosis of HS were reviewed.

### 2.1 Inclusion Criteria

Cats were eligible for inclusion if they met the following conditions:

- Displayed clinical signs consistent with HS, such as licking or biting of the flank/lumbar area, muscle spasms, or rolling skin in the dorsal lumbar, tail, or perianal regions, self‐mutilation or behavioral changes including agitation, sudden vocalization, and aggression.
- Underwent a comprehensive neurological examination performed by either an ECVN resident or diplomate.
- If behavior modification therapy was applied, a comprehensive behavioral assessment was performed by an ECAWBM diplomate.
- Had normal results on complete blood count and serum biochemistry panels.
- Showed no abnormalities on magnetic resonance imaging (MRI) of the lumbar spine and, if performed, on cerebrospinal fluid (CSF) analysis.
- Had a minimum follow‐up period of at least 1 year.

Cats were excluded if they had a history of clearly defined dermatological conditions that could mimic the described signs, lumbar myelopathy, inflammatory or infectious diseases, spinal neoplasms, primary spinal or tail trauma, or intervertebral disc disease.

**FIGURE 1:**
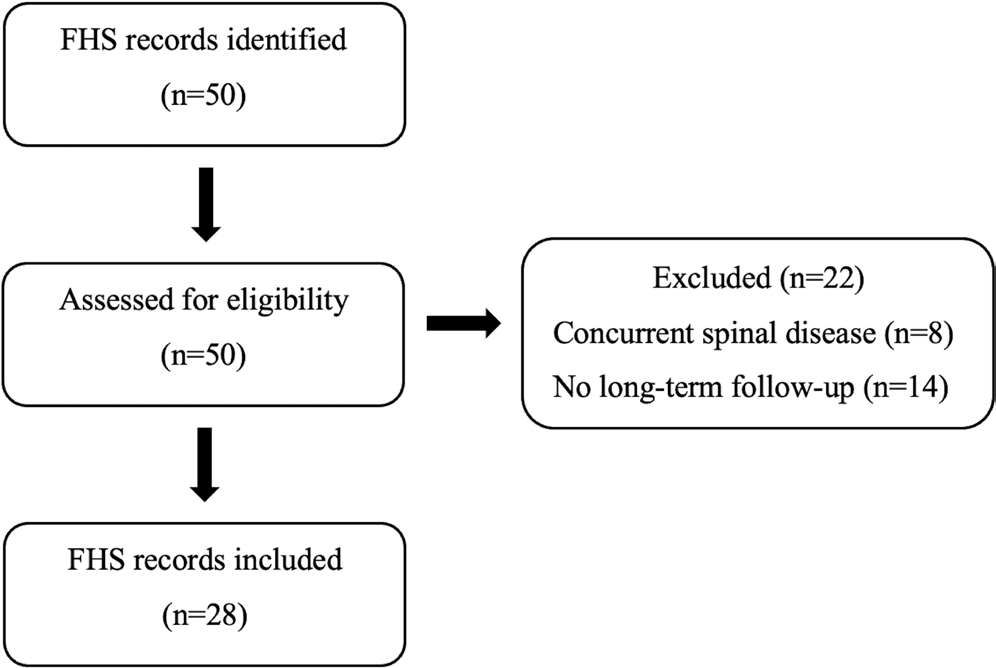
Flowchart of case selection for the study population.

Figure 1 illustrates the selection process of the clinical cases included in the study.

The included cases were divided into three groups based on the different treatment received: a group named Fluoxetine‐only that included cats that received only fluoxetine (without behavioral modification), a group named Fluoxetine/Gabapentin + Behavior that included cats receiving behavioral modification associated with pharmacotherapy, and a group named Behavior‐only that included cats treated with behavioral modification alone (without pharmacotherapy).

Regardless of the severity of clinical signs, all cats allocated to group Fluoxetine‐only received pharmacological treatment alone, as their owners declined behavioral therapy due to financial or logistical constraints. Inclusion in group Behavior‐only was associated with a higher level of owner compliance and willingness to adhere to behavioral therapy as the sole treatment approach.

The recovery time was defined as the number of days from the initiation of therapy to the resolution of clinical signs associated with HS. Recovery was assessed through neurological examinations, complemented by owners' reports confirming the complete absence of HS‐related clinical signs in daily life. The episode‐free period (EFP) was defined as the time between the resolution of clinical signs and the 1‐year follow‐up, during which no recurrence was observed. Signalment, neurological and behavioral alterations, laboratory tests results, MRI and CSF findings, duration of clinical signs, time from clinical signs onset to initiation of therapy, therapy duration, and time to clinical recovery were documented. Additional information concerning adverse effects, therapy adjustments, and the occurrence of relapses within 1 year were also recorded. Caregivers were guided to accurately read and interpret emotional and behavioral responses of cats, with a strict avoidance of punishment‐based methods [11]. Behavioral modification was fundamentally based on the respect of the “5‐Pillars Framework,” which provides a structured approach to environmental assessment and modification, aiming to create an enriched environment that minimizes stress and promotes optimal wellbeing of cats [12]. The five key pillars are as follows: (1) safe space: the provision of secure retreats for refuge and escape; (2) resource availability: access to multiple, spatially separated feeding, litter, and resting areas; (3) play and predation: opportunities for engaging in natural behaviors such as hunting, climbing, and exploring; (4) positive social interaction: encouraging appropriate human–cat interactions and minimizing social stressors; (5) olfactory enrichment: respecting the importance of scent marking and providing opportunities for olfactory stimulation [12].

### 2.2 Statistical Analysis

A descriptive statistical analysis was performed on the overall sample and on each group. Quantitative variables were reported as median and interquartile range (IQR), while qualitative variables were described as absolute and relative frequencies. All statistical analyses were performed using Stata 18 software (StataCorp, 4905 Lakeway Drive College Station, Texas 77845 USA).

## 3 Results

Clinical records of 28 cats diagnosed with HS were included in the study.

Sixteen of them (57%) were assigned to the Fluoxetine‐only group, seven (25%) to the Fluoxetine/Gabapentin + Behavior group, and five (18%) to the Behavior‐only group.

Age at presentation, sex, and breed of the included cats are reported in Table 1.

**TABLE 1:** Descriptive statistics of age at presentation, sex, and breed in the overall sample and in individual groups.

		Groups		
	Overall sample (*n* = 28)	Fluoxetine–only (*n* = 16)	Fluoxetine/Gabapentin + Behavior (*n* = 7)	Behavior‐only (*n* = 5)
Age, years, median (IQR)	3.7 (2.9–5.6)	3 (1.8–5)	6 (3.2–6.7)	3.8 (3.2–9)
Sex, males, *n* (%)	17 (61)	10 (62.5)	6 (86)	1 (20)
Breed				
DSH, *n* (%)	24 (86)	13 (81)	6 (86)	5 (100)
Siberian, *n* (%)	1 (4)	1 (6)	0 (0)	0 (0)
Siamese, *n* (%)	1 (4)	1 (6)	0 (0)	0 (0)
Persian, *n* (%)	1 (4)	1 (6)	0 (0)	0 (0)
Main Coon, *n* (%)	1 (4)	0 (0)	1 (14)	0 (0)

All the enrolled cats exhibited multiple daily episodes of clinical signs consistent with HS. Neurological examinations revealed no abnormalities in any of the cats.

Overall, during the first month of treatment, 24 cats (86%) showed clinical improvement, and 15 of them (54% of the total) experienced complete resolution of clinical signs.

Descriptive statistics for the duration of clinical signs prior to clinical evaluation, the interval between clinical signs onset and initiation of therapy, time to recovery, EFP, therapy modifications, adverse effects, and relapses within 1‐year of treatment initiation, as well as the number of cats without clinical signs and those still receiving pharmacotherapy at the 1‐year follow‐up, in the overall sample and in the three treatment groups are summarized in Table 2.

**TABLE 2:** Descriptive statistics of duration of clinical signs before clinical examination, time interval from clinical signs onset to therapeutic intervention, duration of therapy, time to recovery, occurrence of therapy modification, adverse effects, and relapses within 1 year follow‐up, in the overall sample and in individual groups.

		Groups		
	Overall sample (*n* = 28)	Fluoxetine‐only (*n* = 16)	Fluoxetine/Gabapentin + Behavior (*n* = 7)	Behavior‐only (*n* = 5)
Clinical signs duration, days, median (IQR)	75 (19–345)	35 (7.5–365)	90 (30–270)	270 (240–360)
Time from clinical signs onset to therapy, days, median (IQR)	75 (29–345)	50 (17.5–365)	90 (30–270)	270 (240–360)
Time to recovery, days, median (IQR)	24 (8–90)	8 (3.5–18)	100 (90–210)	60 (30–90)
Pharmacotherapy modification, yes, *n* (%)	11 (39)	8 (50)	3 (43)	0 (0)
Adverse effects, yes, *n* (%)	7 (25)	6 (37.5)	1 (14)	0 (0)
Cases of relapses, yes, *n* (%)	1 (4)	1 (6)	0 (0)	0 (0)
Episode‐free period ≥ 9 months, *n* (%)	23 (82)	15 (94)	4 (57)	4 (80)
Under pharmacotherapy at 1 year, *n* (%)	14 (50)	13 (81)	1 (14)	0 (0)
Absence of clinical signs at 1 year follow‐up, *n* (%)	26 (93)	14 (87.5)	7 (100)	5 (100)

### 3.1 Fluoxetine‐Only Group

All 16 cats included Fluoxetine‐only underwent an MRI scan, and seven of them (44%) a CSF examination. A pharmacotherapy adjustment was needed in eight cats (50%). In four of them (25%), fluoxetine was started at 1 mg/kg every 48 h during the first 7–14 days and subsequently increased to 1 mg/kg once daily. In 12 (75%) cats, fluoxetine was started at 1 mg/kg once daily, but in four of them (25%), it was increased due to insufficient efficacy, reaching 2 mg/kg per day in three cases and 3 mg/kg per day in the other one.

Side effects were described in six (37.5%) cats and consisted of anorexia in all of them, which occurred whenever the dosage was increased. Anorexia was associated with diarrhea in one case and vomiting in another.

At the 1‐year follow‐up, 14 cats (87.5%) were free of clinical signs consistent with HS, 15 cats (94%) had experienced an EFP of at least 9 months, and most of them (81%) remained on pharmacological treatment. Relapse occurred in only one case (6%), which was associated with the discontinuation of pharmacological therapy.

### 3.2 Fluoxetine/Gabapentin + Behavior Group

Five (71%) cats in group Fluoxetine/Gabapentin + Behavior underwent an MRI scan and two (29%) a CSF examination.

Cats in the Fluoxetine/Gabapentin + Behavior group showed more severe clinical signs, including owner‐directed aggression and/or self‐injurious behavior. The pharmacological treatment consisted of gabapentin at 10 mg/kg twice daily in two (29%) and at 10 mg/kg three times daily in three (43%) cats. Additionally, two other animals (29%) were treated with fluoxetine at a dose of 1 mg/kg once daily. One of these two cats began therapy with both gabapentin and fluoxetine. As reported in Table 2, therapy modification was done in three (43%) cats. In one cat, fluoxetine was shifted to gabapentin after 30 days due to excessive agitation. In the other two cases, gabapentin dosage was increased from 10 mg/kg twice to 10 mg/kg three times daily due to insufficient efficacy. Adverse effects were described only in one cat (14%) and consisted of excessive agitation.

At the 1‐year follow‐up, all cats (100%) were free of clinical signs consistent with HS, four cats (57%) experienced an EFP of at least 9 months, and only one cat (14%) remained on pharmacological treatment. No relapses were reported in this group.

### 3.3 Behavior‐Only Group

Two cats (40%) underwent an MRI scan, and no one had a CSF examination.

Cats included in Behavior‐only group exhibited less severe clinical signs compared to Fluoxetine/Gabapentin + Behavior group.

At the 1‐year follow‐up, all cats (100%) were free of clinical signs consistent with HS. Four cats (80%) experienced an EFP of at least 9 months. No relapses were reported during the 1‐year follow‐up period.

## 4 Discussion

The most relevant finding of this retrospective study is that the majority of cats diagnosed with HS, regardless of the therapeutic approach employed, achieved an EFP of at least 9 months and the complete resolution of clinical signs at the 1‐year follow‐up.

Based on these results, we can state that the specific pharmacological and behavioral therapies, whether used alone or in combination, can be considered valid and effective in the management of HS in cats, and that this syndrome can be controlled and treated successfully when approached with targeted interventions.

Most of the cats included in the study had a favorable clinical course. Relapse was documented only in one case during the 1‐year follow‐up, temporally associated with the interruption of pharmacological treatment. Interestingly, all cats treated with behavioral modification and pharmacological therapy did not show relapses after the pharmacological treatment suspension. Furthermore, at the 1‐year follow‐up, most of the cats no longer had HS clinical signs, but the majority of cats belonging to the Fluoxetine‐only group were still undergoing pharmacotherapy, while almost all cases of the Fluoxetine/Gabapentin + behavior group had stopped the drug therapy.

This finding suggests that a behavior modification might control the effects of the trigger factors, allowing the suspension of drug therapy after a few months of treatment. Unfortunately, the retrospective nature of the study did not allow us to evaluate the overall effect of discontinuing therapy in a group treated without behavioral therapy. Therefore, further prospective studies would be fundamental to validate this hypothesis.

Regarding the time required to achieve the resolution of clinical signs, we can argue that behavioral therapy alone or in combination with gabapentin seems to require more time to obtain the remission of the HS clinical signs, compared to the use of fluoxetine alone.

This could be influenced by the longer duration of clinical signs prior to the initiation of therapy, compared to cats treated with fluoxetine alone. Other possible explanations could be that the owner attempts at behavioral modification could make recovery take longer and that gabapentin as add‐on pharmacotherapy might be less rapid or effective in acting than fluoxetine. We can therefore hypothesize that the use of pharmacotherapy, specifically fluoxetine, might be useful in the most severe cases, when a more rapid remission of clinical signs is required or in case of the unavailability of a veterinarian expert in behavioral medicine. So, we can speculate that a multimodal approach, combining pharmacological therapy and behavioral modification, can represent the most effective strategy for cats with HS.

Hyperesthesia syndrome in cats might have a neurobehavioral component, with hypotheses suggesting it arises from either a primary dysfunction within the corticolimbic system (comprising prefrontal cortices, amygdala, and hippocampus) or as a behavioral response to a negative affective state, such as frustration [13]. This system integrates emotion and cognition, leading to flexible behavioral outputs based on the environmental context. Many behavioral disorders in cats arise from disruptions to emotional equilibrium, stemming from perceived or actual threats or the frustration of thwarted natural behaviors [11]. These disruptions can manifest as distress, leading to behavioral changes such as aggression, flight, or attempts to manipulate the olfactory environment (e.g., inappropriate urination and defecation) [14] and probably behavioral pathology. The positive outcomes observed following behavioral therapy strongly support the hypothesis of a significant behavioral component in HS. In contrast to fully domesticated species such as dogs, cats require careful consideration of their environmental and social needs. Potential stressors for indoor cats include restricted access to territory (e.g., confinement within the home), changes in the established household routine, and the presence of other animals. Olfactory cues play a pivotal role in the perception and interpretation of these environmental stressors [15].

Direct social stressors encompass both inter‐ and intra‐household cat conflicts, as well as negative human–cat interactions. The latter might stem from inadequate socialization during critical developmental periods, aversive experiences such as physical punishment, or the development of maladaptive coping mechanisms in response to perceived or actual threats. These raise concerns regarding the potential impact on cats' mental wellbeing. While these concerns are well founded, the magnitude of such impacts remains inadequately quantified, but our results confirm that.

Although HS is a common condition in cats [2, 3, 4], the pathophysiological mechanism and etiology remain unknown. This explains the broad use of various medications in the past. Treatment options such as phenobarbital, gabapentin, prednisolone, amitriptyline, fluoxetine, clomipramine, and multivitamin supplements have been employed, but their responses have been inconsistent or variable [1, 2, 3, 8, 9]. A standardized treatment is still lacking to date.

Furthermore, the long‐term outcomes and treatment options for HS in cats remain underexplored in the literature. However, our findings indicate that any pharmacological intervention should include medications with anxiolytic properties.

The choice of gabapentin arises from its anxiolytic properties and usefulness in the treatment of neuropathic pain. Gabapentin is a structural analogue of the gamma‐aminobutyric acid (GABA) neurotransmitter, used as an anticonvulsant, able to cross the blood–brain barrier. The exact mechanism of action is not completely understood. It has been suggested that gabapentin selectively activates presynaptic GABA‐B heteroreceptors on glutamatergic terminals, while not influencing GABA‐B autoreceptors on GABAergic terminals [16].

It was proposed that gabapentin exerts its effect on the locus coeruleus by inhibiting voltage‐gated calcium channels and increasing extracellular glutamate. This increase in Glu activates the descending noradrenergic system, which projects to the lumbar spinal cord, where the released noradrenaline interacts with α2‐adrenergic receptors to decrease the transmission of nociceptive signals [17].

In cats, gabapentin has been tested as a treatment for anxiety disorders; it can be used without relevant sedative effects to reduce the stress associated with transport to the veterinary clinic [18, 19, 20, 21]. In terms of side effects, occasionally, similar to dogs, ataxia and sedation have also been observed in cats following gabapentin administration [22]. Currently in humans, SSRIs are still regarded as the first‐line treatment for depression [23]. In addition, SSRIs are prescribed for the treatment of obsessive‐compulsive disorders, panic disorder, alcoholism, obesity, and anxiety, as well as various psychological conditions such as migraine headache syndromes and chronic pain [24, 25].

Fluoxetine is employed to manage behaviors such as urine spraying [26], aggression [27, 28], and compulsive actions, including psychogenic alopecia [27, 29, 30]. It is part of the selective serotonin reuptake inhibitors. SSRIs inhibit the reuptake of serotonin, increasing its availability in the synapses and increasing serotonin levels at presynaptic axon terminals [31], with only a minimal effect on the reuptake of norepinephrine; however, the exact action mechanism is not yet fully clear [32, 33]. In cats, some references report adverse effects such as sedation, loss of appetite, gastrointestinal disturbances, irritability, and hyperactivity [34, 35, 36]; adverse effects observed in cats after an overdose even include hyperthermia [37].

In our study the reported adverse effects were mild and involved a minimum number of cats. The most were observed in the Fluoxetine‐only group and seemed to be dose‐related and included mainly anorexia or gastrointestinal signs. Only one cat had a dosage reduction in the drug after 1 week due to the onset of adverse effects. Relapse occurred in only one case; it was associated with the discontinuation of pharmacological therapy.

Given the documented prior use of both gabapentin and fluoxetine in HS cases, and considering their established anxiolytic properties, it can be hypothesized that these pharmacological interventions might also target the psychological dimension of HS.

Of the cats who had a therapeutic adjustment, most only needed an increase in dosage to achieve a more complete clinical response.

The main limitations of this study include its retrospective design, the small sample size, and the heterogeneity of the study groups. These factors limit the inference of our results to the general population of cats with HS and preclude definitive conclusions regarding the superiority of one therapeutic approach over another. However, these preliminary results might serve as a foundation for future prospective, randomized studies designed to compare different treatment strategies in well‐defined, homogeneous populations of cats with HS. Another limitation is the main assessment of resolution of clinical signs by the owners. This method could have failed to detect signs not observed by the owners and could therefore lead to an overestimation of the number of subjects considered to have achieved complete clinical signs remission. Certainly, a 24‐h video recording of the cat's activities would provide more accurate information regarding the presence or absence of HS‐related signs. However, given the impracticality of this approach in clinical settings, owner observations combined with neurological evaluations currently represent the only available method for assessing the presence or absence of clinical signs of HS in cats. Another bias that can influence our results could be that we presumed that cats included in the Behavior‐only group had a less severe HS clinical signs than the Fluoxetine/Gabapentin + Behavior group and do not know exactly the severity of cats belonging to the Fluoxetine‐only group.

In conclusion, the combined approach of pharmacological therapy and behavioral modifications might represent the ideal multimodal approach to treat HS in cats and reach a favorable outcome in the long‐terms [2, 31, 38]. Furthermore, although this study demonstrates that all types of treatments were effective, we can speculate that pharmacological therapy could serve as an initial support during concurrent behavioral modifications, enabling, after achieving effective results through behavioral changes, a faster reduction in the pharmacological therapy until discontinuation. However, prospective studies are necessary to determine which method or therapeutic protocol is most effective for the treatment of HS in cats.

## Disclosure

Authors declare no off‐label use of antimicrobials.

## Ethics Statement

Authors declare no institutional animal care and use committee or other approval was needed. Authors declare human ethics approval was not needed.

## Conflicts of Interest

The authors declare no conflicts of interest.
